# Small RNA Sequencing Reveals a Distinct MicroRNA Signature between Glucocorticoid Responder and Glucocorticoid Non-Responder Primary Human Trabecular Meshwork Cells after Dexamethasone Treatment

**DOI:** 10.3390/genes14112012

**Published:** 2023-10-27

**Authors:** Kandasamy Kathirvel, Xiaochen Fan, Ravinarayanan Haribalaganesh, Devarajan Bharanidharan, Rajendrababu Sharmila, Ramasamy Krishnadas, Veerappan Muthukkaruppan, Colin E. Willoughby, Srinivasan Senthilkumari

**Affiliations:** 1 Department of Ocular Pharmacology, Aravind Medical Research Foundation #1, Anna Nagar, Madurai 625020, Tamilnadu, India; 2 Department of Bioinformatics, Aravind Medical Research Foundation, Madurai 625020, Tamilnadu, India; bharani@aravind.org; 3 Institute of Life Course and Medical Sciences, University of Liverpool, Liverpool L69 7ZX, UK; xiaochenfan.emily@gmail.com; 4 Glaucoma Clinic, Aravind Eye Hospital, Madurai 625020, Tamilnadu, Indiakrishnadas@aravind.org (R.K.); 5 Aravind Medical Research Foundation, Madurai 625020, Tamilnadu, India; muthu@aravind.org; 6 Genomic Medicine, Biomedical Sciences Research Institute, Ulster University, BT52 1SA Coleraine, UK

**Keywords:** glucocorticoids, glaucoma, microRNAs, miRNA, trabecular meshwork, small RNA-Seq, pathway analysis, ocular hypertension, intra-ocular pressure

## Abstract

Glucocorticoids (GCs) are known to regulate several physiological processes and are the mainstay in the management of inflammatory eye diseases. The long-term use of GC causes raised intraocular pressure (IOP) or ocular hypertension (OHT) in about 30–50% of the susceptible individuals depending on the route of administration, and can lead to steroid-induced secondary glaucoma. The present study aims to understand the role of microRNAs (miRNAs) in differential glucocorticoid (GC) responsiveness in human trabecular meshwork (HTM) cells using small RNA sequencing. The human organ-cultured anterior segment (HOCAS) model was used to identify whether donor eyes were from GC-responders (GC-R; *n* = 4) or GC-non-responders (GC-NR; *n* = 4) following treatment with either 100 nM dexamethasone (DEX) or ethanol (ETH) for 7 days. The total RNA was extracted from cultured HTM cells with known GC responsiveness, and the differentially expressed miRNAs (DEMIRs) were compared among the following five groups: Group #1: ETH vs. DEX-treated GC-R; #2: ETH vs. DEX-treated GC-NR; #3: overlapping DEGs between Group #1 and #2; #4: Unique DEMIRs of GC-R; #5: Unique DEMIRs of GC-NR; and validated by RT-qPCR. There were 13 and 21 DEMIRs identified in Group #1 and Group #2, respectively. Seven miRNAs were common miRNAs dysregulated in both GC-R and GC-NR (Group #3). This analysis allowed the identification of DEMIRs that were unique to GC-R (6 miRNAs) and GC-NR (14 miRNAs) HTM cells, respectively. Ingenuity Pathway Analysis identified enriched pathways and biological processes associated with differential GC responsiveness in HTM cells. This is the first study to reveal a unique miRNA signature between GC-R and GC-NR HTM cells, which raises the possibility of developing new molecular targets for the management of steroid-OHT/glaucoma.

## 1. Introduction

Glucocorticoids (GCs) are known to regulate several physiological processes and are the mainstay in the management of systemic and ocular autoimmune and inflammatory eye diseases [1]. Topical steroids are being prescribed to reduce inflammation following cataract surgery and the treatment duration ranges from a few weeks to a year or longer [2]. It is shown that up to 35% of the patients without a prior glaucoma diagnosis and up to 80% of those with pre-existing glaucoma experience clinically significant post keratoplasty intraocular pressure (IOP) elevation with long-term topical glucocorticoid use [2].

Intravitreal steroids, such as intravitreal triamcinolone acetonide (IVTA), dexamethasone (DEX) intravitreal insert, and fluocinolone acetonide implant, have been in use in ophthalmology to treat posterior segment inflammatory eye disorders, such as diabetic macular edema, non-infectious uveitis, and retinal vein occlusion [3,4,5,6]. The onset and duration of ocular hypertension (OHT) or raised IOP after intravitreal administration of steroids varies according to the molecule and dose. With 1 mg IVTA, the median number of days since first injection to IOP elevation greater than 10 mmHg from baseline was 34 d and 52.5 d for the patients treated with 4 mg IVTA [7]. In case of non-biodegradable intravitreal fluocinolone acetonide, the onset of OHT began within 2–4 weeks with a maximum at 24 weeks and returned to a basal values in 9–12 months after implantation [8,9]. The time course of OHT for dexamethasone implantation was in an interval of 1.5–2.5 months [10]. All these studies emphasized that moderate to severe IOP elevation is associated with intravitreal steroid use, which warrants the close monitoring of patients. Hence, the steroid response represents a major clinical challenge and the ability to predict the risk of steroid glaucoma, and tailor monitoring, and potentially treatment dose to individual patients is an attractive goal to deliver personalized medicine.

Steroid-induced OHT, if left untreated, can lead to secondary open-angle glaucoma [11]. Individuals with the risk of developing elevated IOP (GC responders) following steroid use are more likely to develop the most prevalent form of glaucoma, i.e., primary open-angle glaucoma (POAG); furthermore, patients with pre-existing glaucoma show more than 90% susceptibility to steroid-induced raised IOP [12,13]. The general population could be divided into the following three groups based on their response to topical steroid administration: [14] high responders, 4–6% of the population, developed an IOP ≥ 31 mmHg or ≥15 mmHg above baseline [15]; moderate responders, approximately one-third of the population, had IOPs between 20 and 31 mmHg, or a pressure rise of 6–15 mmHg; and [16] non-responders, the remaining two thirds, had pressure increases ≤6 mmHg and IOPs ≤ 20 mmHg. There have been limited studies investigating the genetic basis of the steroid response. In some patients, glucocorticoid receptor gene mutations might influence the IOP elevation response, but the evidence for this is not clear [17,18]. Even though POAG and steroid-induced glaucoma share similar clinical presentations, the molecular and genetic mechanisms responsible for the differential GC responsiveness in individuals are not well understood. Given that no genetic markers allow for the identification of GC, responders our lab has previously used the human organ-cultured anterior segment (HOCAS) model to determine the GC responsiveness of donor eyes for studies into the molecular basis of the steroid response [19,20] as previously described.

MicroRNAs (miRNAs) are small non-coding RNAs, which regulate gene expression by either mRNA degradation or translational repression [21]. MiRNAs have been detected in most of the biological fluids where they are preserved in extracellular vesicles, or bound to carrier proteins thereby providing a remarkable stability to miRNAs [22]. These properties makes miRNAs suitable bio-markers for many diseases including ocular diseases [22,23,24,25]. In the eye, miRNAs are expressed in tissue-specific fashion and have a specific role in ocular development and retinal homeostasis [26,27]. MiRNAs have been identified in ocular fluids such as tears, aqueous humor, and vitreous humor [24]. Several studies documented the expression of glaucoma-associated miRNAs in the affected fluids/tissues such as aqueous humor, tears, trabecular meshwork (TM), and retina of patients with glaucoma and animal models [24,28,29,30,31]. The miRNA expression profile of the TM in response to patho-physiologically relevant stressors, such as cyclic mechanical stress, oxidative stress, and stress-induced premature senescence, has been reported and contribute to vital cellular functions [32,33,34,35,36].

GC production by the adrenal glands and GC-mediated cellular response are also regulated by miRNAs [37]. In the TM, dexamethasone (DEX) treatment induces cellular and extracellular remodeling leading to an increased outflow resistance and elevated IOP [38]. The pharmacological actions of GCs are mediated by the glucocorticoid receptor whose activation results in the stimulation of target gene expression that regulates several complex signaling pathways [39,40]. The contribution of miRNA in the regulation of GC activity and signaling, which results in the differential GC responsiveness in the TM is currently unknown. Therefore, in this study the differential expression of miRNAs in primary HTM cells with known GC responsiveness was investigated using small RNA sequencing. A unique miRNA signature between GC-R and GC-NR HTM cells was identified and understanding the role of miRNAs in GC responsiveness raises the possibility of new molecular targets for the management of steroid-induced OHT/glaucoma.

## 2. Materials and Methods

### 2.1. Human Donor Eyes

Post-mortem human cadaveric eyes not suitable for corneal transplantation were obtained from the Rotary Aravind International Eye Bank, Aravind Eye Hospital, Madurai. The study was conducted following approval from the Human Ethics Committee of the Institute. The tissues were handled in accordance with the Declaration of Helsinki. The donor eyes were enucleated within 5 h of death (mean elapsed time between death and enucleation was 2.75 ± 1.58 h) and kept at 4 °C in the moist chamber until culture. All eyes were examined under the dissecting microscope for any gross ocular pathological changes and only macroscopically normal eyes were used for the experiments. The characteristics of donor eyes used for this study are summarized in Supplementary Table S1.

### 2.2. Primary Human Trabecular Meshwork (HTM) Cells with Known GC Responsiveness

In a set of paired eyes, one eye was used to establish HOCAS ex vivo model system to characterize GC responsiveness after DEX treatment as previously described by our group [19]. The other eye was used to establish primary HTM cultures from eyes with identified GC responsiveness [40]. The TM tissue was excised from the other eye of each set of paired eyes and primary HTM cell culture was established by extracellular matrix digestion method as described previously [41,42]. Primary HTM cells were grown at 37 °C in 5% CO_2_ in low glucose Dulbecco’s modified Eagle medium (DMEM-Low glucose; Gibco, Grand Island, NE, USA) with 15% fetal bovine serum (Gibco, Grand Island, NE, USA), 5 ng/mL basic fibroblast growth factor and antibiotics. The primary HTM cells isolated from the other eye of each pair were characterized with aquaporin (Santa Cruz Biotechnology, Dallas, Oregon, USA), myocilin (R&D Systems, Minneapolis, MN, USA) and phalloidin (Vector Laboratories, Newark, CA, USA) staining by immunofluorescence analysis using confocal microscope (Leica, Wetzlar, Germany). HTM cells with more than 50% myocilin positivity in response to DEX treatment were used for further experiments [43]. Confluent cultures of GC-R and GC-NR HTM cells were then treated with either 100 nM DEX or 0.1% ethanol (ETH) as a vehicle control over a period of 7 days and the medium was exchanged every other day (3 doses of DEX treatments). HTM cells from passages 2–4 were used for all experiments. At the end of DEX or 0.1% ETH treatment for 7 days, HTM cells from each GC-R (*n* = 4) and GC-NR (*n* = 4) HTM cells were subjected to RNA extraction and small RNA sequencing.

### 2.3. Total RNA Extraction

Total RNA including miRNA was extracted from GC-R and GC-NR HTM cells post 7d DEX treatment using the TRIzol reagent. Briefly, 200 µL of chloroform was added to 1 mL of TRIzol containing one million HTM cells. Then, 500 µL of isopropyl alcohol and 1 µL of glycogen were added into RNA containing aqueous phase for nucleic acid precipitation after centrifugation at 13,000 rpm for 15 min at 4 °C (Megafuge 8R, Thermo Scientific, Osterode am Harz, Germany). RNA pellet was washed twice with 75% ethanol and eluted with 20 µL of nuclease free water. RNA quantity and quality were assessed by the ratio of absorbance at 260/280 nm using NanoDrop 2000 spectrophotometer (Thermofisher Scientific, Wilmington, UK), and TapeStation (Agilent Technologies, Santa Clara, CA, USA), respectively.

### 2.4. Library Preparation and miRNA Sequencing

MiRNA sequencing libraries were prepared using NEBNext smallRNA library prep kit (NEB, Ipswich, MA, USA). Briefly, RNA fragments of different sizes were separated by PAGE and 18 to 30 nt stripe was ligated with specific adapters at 3′ and 5′ end. Then, libraries were reverse transcribed followed by PCR amplification. The final enriched libraries were purified and quantified by Qubit (ThermoFisher Scientific, Mumbai, India) and the size was analyzed by Bioanalyzer (Agilent, Bangalore, India). Individual libraries were pooled with specific index sequences and then sequenced by Illumina Next Seq 500 platform (Illumina, Inc., San Diego, CA, USA), according to the manufacturers protocol. Around 10 million reads were generated from each HTM donor RNA sample and obtained through de-multiplexing.

### 2.5. Differential Expression Analysis of miRNAs

The quality of raw data of miRNA sequencing was assessed by FastQC tool(ver.23.2). The PCR duplicates and low-quality reads were excluded using cutadapt3 in the data cleaning step. The pre-processed high quality miRNA reads were then mapped with human reference genome GRCh38 using STAR aligner with default parameters. The expression of miRNAs in read count was obtained from individual aligned BAM files using FeatureCounts with miRbase annotation (release 22). MiRNAs with less than five read counts were excluded for further analysis. TMM (timed mean of M values) strategy was employed for data normalization and differential expression was performed using EdgeR, package from Bioconductor. The miRNAs were considered as differentially expressed if the absolute fold change (log2) value was more than 1.5, and the *p*-value < 0.05. For comparison, the differentially expressed miRNAs were segregated into 5 groups as described previously [20]. Group #1 = DEMirs between DEX and ETH-treated GC-R HTM cells; Group #2 = DEMirs between DEX and ETH-treated GC-NR HTM cells; Group #3 = commonly expressed miRNAs between Group #1 and Group #2; Group #4 = uniquely expressed miRNAs in GC-R HTM cells (Group #4 = Group #3 minus Group #1); Group #5 = uniquely expressed miRNAs in GC-NR HTM cells (Group #5 = Group #3 minus Group #2).

### 2.6. Validation of DEMirs by Real-Time PCR

The expression of significantly altered miRNAs identified by small RNA sequencing analysis was further validated by RT-qPCR with additional, independent biological replicates of GC-R and GC-NR (*n* = 5 each) HTM cells. Briefly, the total RNA was extracted as described previously [20] and miRNAs from each HTM cells was reverse transcribed into cDNA using miScript II RT kit as per the manufacturer’s instruction (Qiagen, Hilden, Germany). RT-qPCR was performed in a total volume of 20 µL containing 25 ng of total cDNA, 5X SYBR Green master mix and final concentration of primers. The assay was performed on an ABI-QuantStudio 5 (Applied Biosystems, Woburn, MA, USA). All miRNA levels were measured at CT threshold levels and normalized with the average CT values of reference target RNU6. Values were expressed as fold increase over the corresponding values for control by the 2^−ΔΔCT^ method. All PCR reactions were performed in triplicates.

### 2.7. Prediction of the Target mRNA with Their Enriched Pathways and Biological Processes

The target mRNAs of DEMirs were predicted using Ingenuity Pathway Analysis (IPA, Qiagen, Manchester, UK). The DEMirs from all groups (Group #1 to #5) with *p*-value < 0.05 were analyzed using the ‘miRNA Target Filter’ tool in the IPA software based on the TargetScan, TarBase, miRecords contents and Ingenuity^®^ Knowledge Base (IPA, Qiagen, Manchester, UK). The cumulative weighted context score (CWCS) defined by TargetScan was used for assigning different confidence levels of the predicted target mRNAs. The target mRNAs with ‘experimentally observed’ and ‘high confidence level’ were selected into the ‘Target MRNA List 1’.

At the time of miRNA extraction using the same experimental set-up mRNA was also extracted. This provided paired mRNA-seq and miRNA-seq data from the same cells of known GC responsiveness. The mRNA-seq was previously published by our group [20]. The DEMirs from Group #1 to #5 were further therefore combined with our previously generated mRNA library [20] to find the negatively correlated miRNA targets (Target MRNA List 2) using the IPA software in paired datasets from the same TM cell line donors. The prediction of the pathways and biological processes enriched in the ‘Target MRNA List 1’ and ‘Target MRNA List 2’ was performed using the GSEApy package/python (v 3.8.3) (for ‘Target MRNA List 1’ without logFC and *p*-values) [44] and/or IPA software (for ‘Target MRNA List 2’ with logFC and *p*-values).

### 2.8. Statistical Analysis

Statistical analysis was carried out using Graph Pad Prism (ver.8.0.2) (Graph Pad software, San Diego, CA, USA). All data are presented as mean ± SEM or otherwise specified. Statistical significance between two groups was analyzed using unpaired 2-tailed Student’s t test. *p* < 0.05 or less was considered as statistically significant.

## 3. Results

### 3.1. Establishment of GC-R and GC-NR HTM Cells

In this study, one eye of each set of the paired eyes was used to assess the GC responsiveness in HOCAS after 100 nM DEX treatment for 7 days and the other eye was used to establish the primary HTM cells. Based on the IOP response, the HTM cells established from each donor eye were categorized as GC-R or GC-NR cells [19,20]. The details of the human donor eyes used in this study are summarized in Supplementary Table S1.

### 3.2. miRNA-Seq Data Quality

In total, from 9.1 to 17.2 million reads were generated from miRNA sequencing for each primary HTM cells. Pre-aligned QC reports showed the quality score (Phred or Q score) of miRNA reads were ≥30. An average of 97% of the reads from miRNA-seq data were aligned with human reference genome GRCh38. The details of miRNA-sequencing and alignment statistics are shown in Supplementary Table S2. EdgeR with Benjamini–Hochberg corrections was used to identify the differentially expressed miRNAs from all five groups (Table 1; Supplementary Table S3a,b).

### 3.3. Differentially Expressed Genes of GC-R and GC-NR HTM Cells

**Figure 1:**
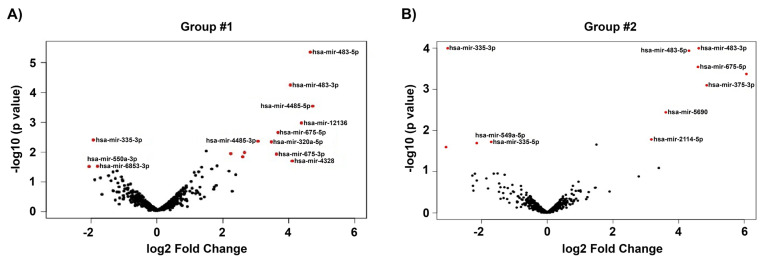
Volcano plot showing the distribution of DE miRNAs. The dys-regulated DEMirs of GC-responder and non-responder HTM cells are shown in (**A**,**B**) respectively. The fold of change (log2) and *p*-value (−log 10) of the dys-regulated miRNAs in DEX-treated cells compared to vehicle control are shown in volcano plot. Significant *p*-value < 0.05, only taken for consideration (red color).

**Figure 2:**
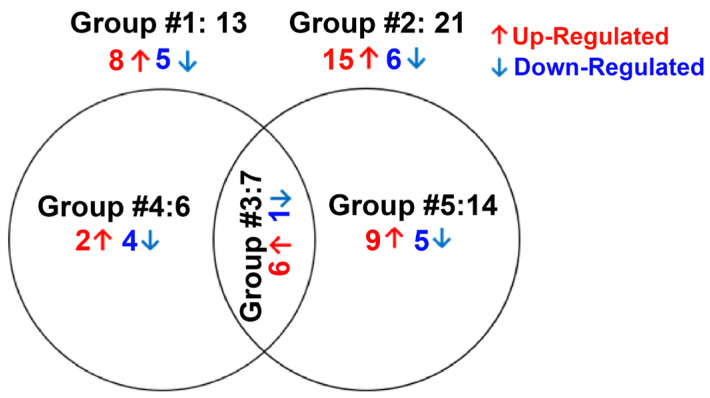
Venn diagram showing differentially expression groupings. DE miRNAs of three groups from miRNA-seq data are shown. Only genes with absolute fold change 1.5 and significant *p*-value < 0.05 were included in these groupings. Group #1: DE miRNAs between ETH- and DEX-treated cells of GC-R HTM cells, Group #2: DE miRNAs between ETH- and DEX-treated cells of GC-NR HTM cells, Group #3: Overlapping DE miRNAs between Group #1 and Group #2; Group #4: uniquely expressed miRNAs in GC-R and Group #5: uniquely expressed DE miRNAs in GC-NR.

The total number of miRNAs identified in HTM cells of each donor eye ranged from 718 to 898. The expression of DE-miRNAs from GC-R (Group #1) and GC-NR (Group #2) HTM cells are represented in the volcano plot (Figure 1). In total, there were 13 and 21 miRNAs identified as differentially expressed in Group #1 (8 up-regulated; 5 down-regulated) and Group #2 (15 up-regulated; 6 down-regulated), respectively. Seven miRNAs were found as common miRNAs dysregulated in both GC-R and GC-NR (Group #3) with an absolute fold change (log2) value of 1.5, and a *p*-value < 0.05. In total, 6 (2 up-regulated; 4 down-regulated) and 14 (9 up-regulated; 5 down-regulated) miRNAs were found to be uniquely expressed only in GC-R (Group #4) and GC-NR (Group #5) HTM cells, respectively (Figure 2). In Group #4 (GC-R), hsa-miR-2114-3p (log FC = 6.07), hsa-miR-2114-5p (log FC = 3.17) were significantly up-regulated and hsa-miR-335-5p (log FC= −1.71), hsa-miR-549a-5p (logFC= −2.15), hsa-miR-7151-3p (logFC = −3.08) and hsa-miR-124-3p (logFC = −6.21) were significantly down-regulated. Whereas in Group #5 (GC-NR), hsa-miR-4485-5p (logFC = 4.73), hsa-miR-12136 (logFC = 4.39), hsa-miR-4328 (logFC = 4.11) were significantly up-regulated and hsa-miR-181b-2-3p (logFC= −1.21), hsa-miR-486-3p (logFC = −1.34) and hsa-miR-6853-3p (logFC = −1.80) were significantly down-regulated. In Group #3, the commonly expressed miRNAs between Group #1 and #2 were hsa-miR-675-3p, hsa-miR-483-3p, hsa-miR-675-5p, hsa-miR-483-5p, hsa-miR-5690, hsa-miR-6842-3p (up-regulated); and hsa-miR-335-3p (down-regulated). The DEMirs from Group #1 to Group #5 are shown in the Venn diagram (Figure 2) and Table 1 and Supplementary Table S3a,b.

### 3.4. Validation of DE-miRNAs by qPCR

**Figure 3:**
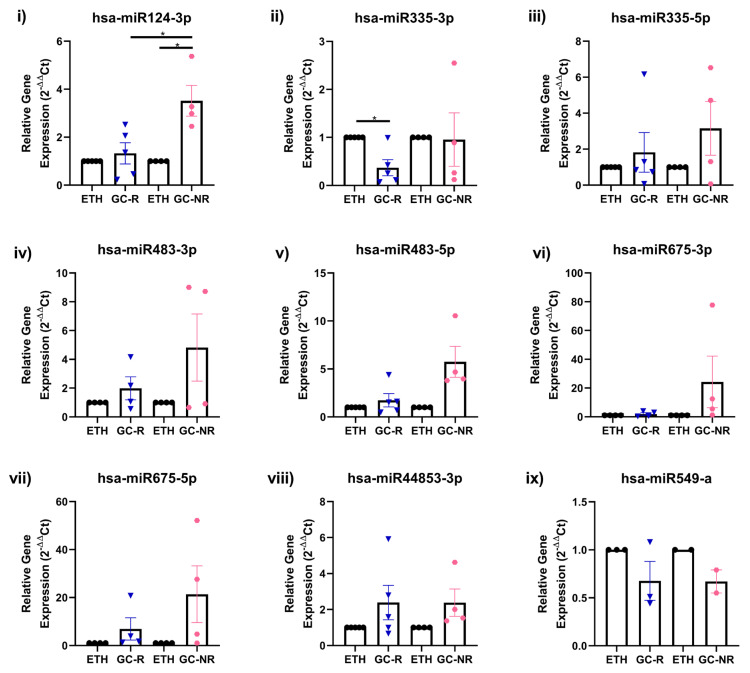
Validation of DE miRNAs by qPCR. Expression profile of selected miRNAs identified from miRNA-seq was validated by qPCR is shown [(i) hsa-miR 124-3p; (ii) hsa-miR 335-3p; (iii) hsa-miR 335-5p; (iv) hsa-miR483-3p; (v) has-miR 483-5p; (vi) hsa-miR 675-3p; (vii) hsa-miR 675-5p; (viii) hsa-miR 44853-3p and (ix) hsa-miR 549-a]. Primary HTM cells were treated with 100 nM DEX or 0.1% ETH for 7 days. Total RNA was extracted, converted to cDNA and the expression profile of selected miRNAs were carried out qPCR. miRNA expressions were normalized to RNU6 and analyzed using the 2^−ΔΔCT^ method [* *p* < 0.05].

**Figure 4:**
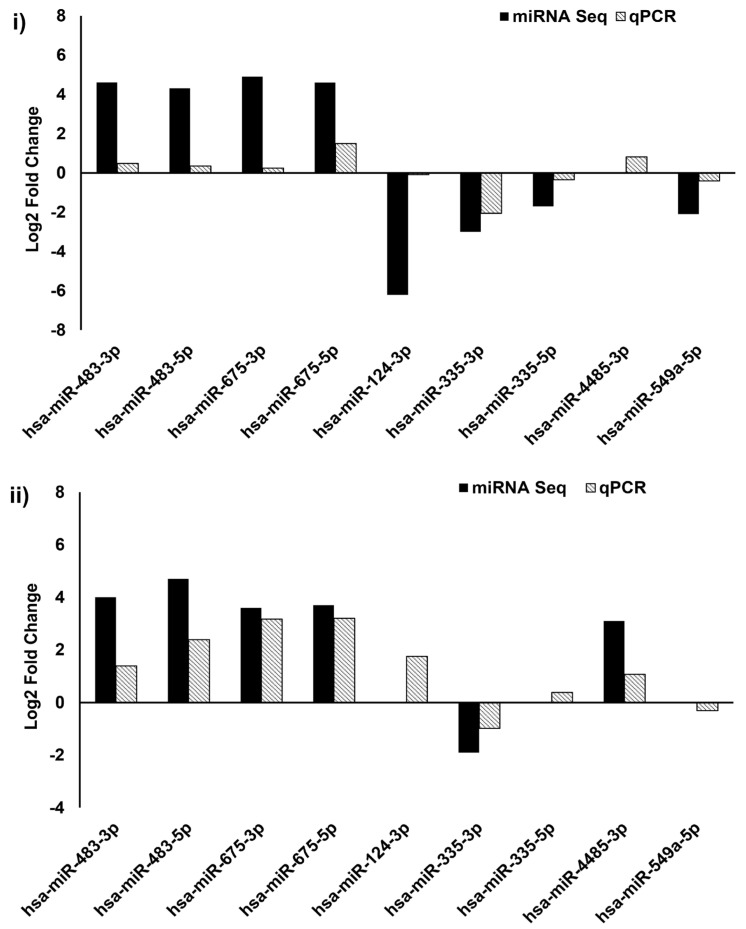
Validation of miRNA sequencing by qPCR. Comparison of selected genes expression profile from Group #1 (**i**) and Group #2 (**ii**) miRNA sequence by qPCR; 2^ΔΔCt^ method was used for calculation of miRNA expression changes and U6 was used as an internal control.

Out of nine miRNAs selected for qPCR (Supplementary Table S5), the expression pattern of seven miRNAs matched with miRNA-seq data (Figure 3 and Figure 4).

#### Prediction of Target mRNAs and Pathways Analysis

(A) Prediction of target mRNAs and pathways in silico analysis- ‘Target MRNA List 1’

**Figure 5:**
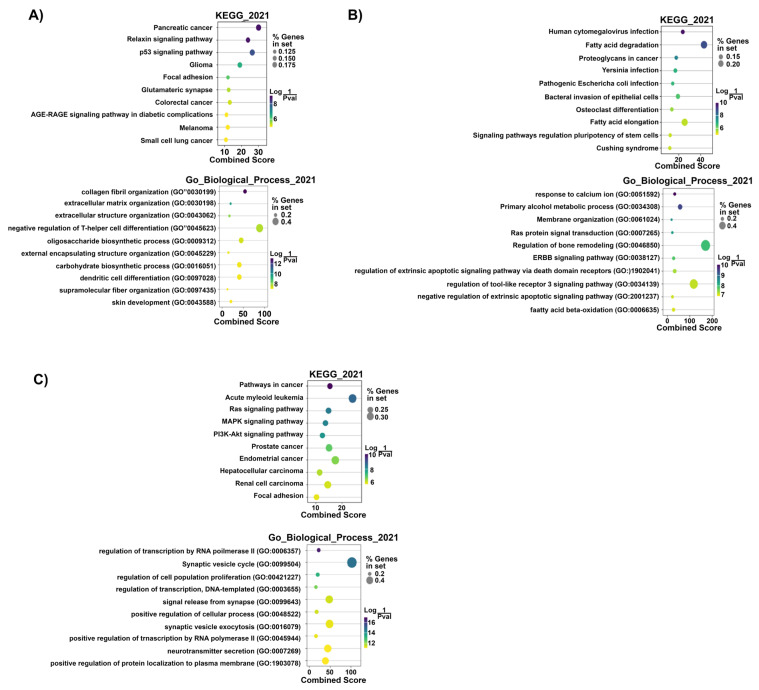
Top 10 predicted KEGG pathways and GO biological processes of the ‘Target MRNA List 1’ of Group #3 (**A**), #4 (GC-R) (**B**), and #5 (GC-NR) (**C**). Dot colors: Log (1/*p*-value). Dot sizes: % genes in set. The combined score is defined based on the percentages of genes in set and Log (1/*p*-value) by [3]. *p*-value < 0.05 was considered to be statistically significant.

The prediction of the target mRNAs in the ‘Target MRNA List 1’ (without logFC and *p*-values) was performed by the IPA software based on the TargetScan, TarBase, miRecords contents, and Ingenuity^®^ Knowledge Base. We found 1354 target mRNAs (74 of 1354 miRNA: mRNA interactions were experimentally validated) in Group #3, 1173 target mRNAs (237 of 1173 miRNA: mRNA interactions were experimentally validated) in Group #4 (GC-R), and 3187 target mRNAs (5 of 3187 miRNA: mRNA interactions were experimentally validated) in Group #5 (GC-NR) (Supplementary File S1 List S1A). The enriched pathways and biological processes in the ‘Target MRNA List 1’ were predicted using the GSEApy package/python (v 3.8.3) [44] based on the KEGG_2021 and GO_Biological_Process_2021 datasets. We found 27 significant pathways and 266 biological processes in Group #3, 46 significant pathways and 360 biological processes in Group #4 (GC-R), and 47 significant pathways and 445 biological processes in Group #5 (GC-NR) (*p*-value < 0.05) (Supplementary File S1 List S1B,C). The top 10 KEGG pathways and biological processes in Groups #4 and #5 based on the smallest *p*-value are shown in Figure 5.

(B) Prediction of target mRNAs that negatively correlated with DEMirs, their pathways and biological processes in experimental analysis—‘Target MRNA List 2’

**Figure 6:**
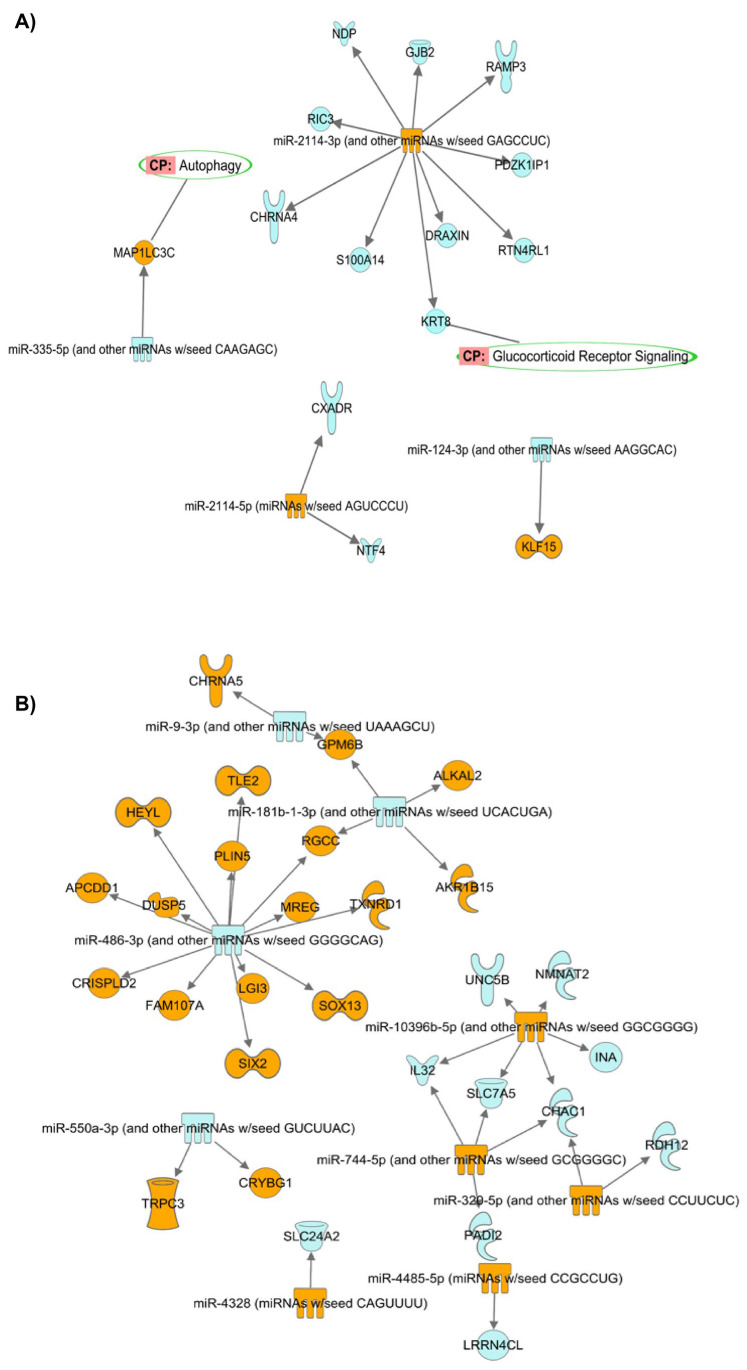
Interaction networks of the DEMIRs from Group #4 (GC-R) (**A**) and Group #5 (GC-NR) (**B**) (absolute LogFC > 2 and *p* < 0.05) and their negatively corelated target mRNAs (absolute FC > 2 and *p* < 0.05). Orange: up-regulated miRNAs/mRNAs. Blue: down-regulated miRNAs/mRNAs. Green circle: predicted pathways that the target mRNAs are located.

The target mRNAs that negatively correlated with the DEMIRs ‘Target MRNA List 2’ (with logFC and *p*-values) were predicted by the IPA software based on our previously generated mRNA library [20]. We found that 2 target mRNAs were negatively correlated with 2 DEMirs in Group #3, 15 target mRNAs were negatively correlated with 4 DEMirs in Group #4 (GC-R), and 12 target mRNAs were negatively correlated with 6 DEMirs in Group #5 (GC-NR) (Table 2). The confidence levels of each predicted target mRNAs are shown in Supplementary File S2 List S2A. The interaction networks of the DEMirs from Group #4 (GC-R) and #5 (GC-NR) and their negatively correlated mRNAs were shown in the Figure 6. The pathway prediction of the ‘Target MRNA List 2’ was performed using the GSEApy package /python (v 3.8.3) (Supplementary File S2 List S2B) and IPA software (Supplementary File S2 List S2C). The GSEApy pathway analysis results of ‘Target MRNA List 2’ (Supplementary File S2 List S2B) were compared to that of ‘Target MRNA List 1’ (Supplementary File S1 List S1B) for finding the overlapping pathways. We found the MAPK signaling pathway was statistically significant in ‘Target MRNA List 1 & 2’ of Group #3 (*p*-value < 0.05). The nicotinate and nicotinamide metabolism pathway were statistically significant both in ‘Target MRNA List 1 & 2’ of Group #5. No significant overlapping pathway was found in the ‘Target MRNA List 1 & 2’ of Group #4.

Table 2 shows the IDs and counts of the target mRNAs negatively correlated with the DEMIRs in Group#3-#5. RGS16: regulator of G protein signaling 16; NGFR: nerve growth factor receptor; KLF15: KLF transcription factor 15; CHRNA4: cholinergic receptor nicotinic α 4 subunit; DRAXIN: dorsal inhibitory axon guidance protein; GJB2: gap junction protein β 2; JCHAIN: joining chain of multimeric IgA and IgM; KRT8: keratin 8; NDP: norrin cystine knot growth factor NDP; PDZK1IP1: PDZK1-interacting protein 1; RAMP3: receptor activity modifying protein 3; RIC3: RIC3 acetylcholine receptor chaperone; RTN4RL1: reticulon 4 receptor like 1; S100A14: S100 calcium binding protein A14; CXADR: CXADR Ig-like cell adhesion molecule; NTF4: neurotrophin 4; MAP1LC3C: microtubule-associated protein 1 light chain 3 γ; CHAC1: ChaC glutathione-specific γ-glutamylcyclotransferase 1; INA: internexin neuronal intermediate filament protein α; NMNAT2: nicotinamide-nucleotide adenylyltransferase 2; UNC5B: Unc-5 netrin receptor B; ALKAL2: ALK and LTK ligand 2; SLC24A2: solute carrier family 24 member 2; DUSP5: dual-specificity phosphatase 5; LGI3: leucine rich repeat LGI family member 3; MREG: melanoregulin; SOX13: SRY-Box transcription factor 13; IL32: interleukin 32; and SLC7A5: solute carrier family 7 member 5.

**Figure 7:**
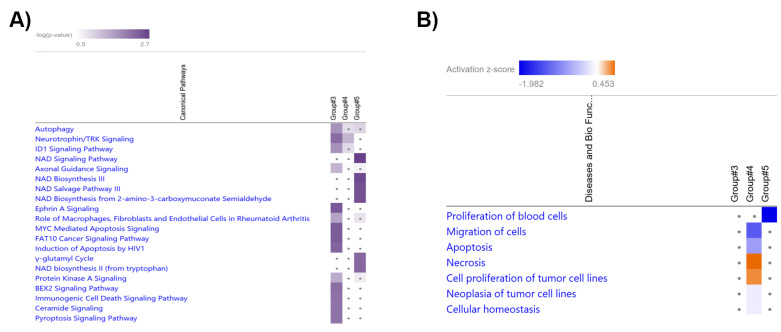
Heatmap shows the top 20 significant pathways (**A**) and biological processes (**B**) in the comparative analysis of Groups #3, #4 (GC-R), and #5 9GC-NR).

The comparative analysis of the pathways of the ‘Target MRNA List 2’ in Group #3, #4 and #5 was performed by IPA software (Supplementary File S2 List 2D). The top 20 significant pathways in Group #3, #4 and #5 ranked by −log(*p*-value) are shown in Figure 7A. The results show that neurotrophin/TRK signaling was significant in Group #3 (−log(*p*-value) = 2.18) and #4 (GC-R) (−log(*p*-value) = 1.34). NAD signaling pathway (−log(*p*-value) = 2.67), NAD biosynthesis III (−log(*p*-value) = 2.55), NAD salvage pathway III (−log(*p*-value) = 2.49), NAD biosynthesis from 2-amino-3-carboxymuconate semialdehyde (−log(*p*-value) = 2.49), NAD biosynthesis II (from tryptophan) (−log(*p*-value) = 2.22), and γ-glutamyl cycle (−log(*p*-value) = 2.22) pathways were found to be significant only in Group #5 (GC-NR) (Supplementary File S2 List S2D). 

The biological process prediction of the ‘Target MRNA List 2’ was performed using the GSEApy package/python (v 3.8.3) and IPA software (Supplementary File S2 List S2E). The comparative analyses of the GSEApy biological processes of the ‘Target MRNA List 1 & 2’ were performed using Python (v 3.8.3). The overlapping biological processes in the ‘Target MRNA List 1 & 2’ of Groups #3–#5 are shown in Supplementary Table S6. The biological processes of the ‘Target MRNA List 2’ were further analyzed and compared by IPA software. The comparative analysis of the biological processes in Group #3, #4, and #5 are shown in Figure 7 (B) (ranked by absolute z-score. z-score is to ascertain the activation states of involved biological functions; z > 0: increased and z < 0: decreased)). The results show that the cell migration (Z-score = −1.295), apoptosis (Z-score = −0.765), and cellular homeostasis (Z-score = −0.147) related biological processes were predicted to be down-regulated in Group #4 (GC-R), while necrosis related biological process (Z-score = 0.453) were up-regulated in Group #4 (GC-R) compared to Group #5 (GC-NR).

## 4. Discussion

Steroid-induced OHT and glaucoma are serious consequences associated with the long-term use of steroids, with many ocular conditions requiring chronic steroid administration [45]. Steroid-induced elevated IOP is due to the increased resistance in the aqueous outflow pathway in the TM. Alterations in the function of TM can eventually lead to increased outflow resistance and elevated IOP [46]; however, the molecular mechanism responsible for the pathogenesis of SI-OHT/SIG is poorly understood [45,47]. Therefore, in the present study, the role of miRNAs in mediating differential GC responsiveness in HTM cells was investigated.

In the trabecular meshwork, key miRNAs have been identified in TM cells from rodent and human subjected to cyclic mechanical stress, ROS, or senescence [33,34,35,36,48]. The identified miRNAs were found to have a crucial role in various cellular processes, such as autophagy, apoptosis, senescence, and neuro-inflammation pathways [33,34,35,36,48].

In TM cells, steroids are known to induce alterations in its structure and function, including the inhibition of cell proliferation and migration [49], cytoskeletal rearrangement (formation of cross-linked actins (CLANs) [50], increased TM cell and nuclear size [51], accumulation of excessive extracellular matrix [52,53], decreased phagocytosis [54], and alterations in cellular junctional complexes [55]. These cellular, biochemical, and morphological changes ultimately lead to an increased outflow resistance and, hence, elevated IOP; however, the expression of miRNAs in the TM in response to steroids has not yet been reported. Therefore, in the present study, the expression of miRNAs in GC-R and GC-NR HTM cells upon DEX treatment was investigated using small RNA sequencing technology.

Our study identified a unique miRNA signature between GC-R and GC-NR HTM cells. Specifically, 6 miRNAs were differentially expressed in GC-R HTM cells, and 14 miRNAs were differentially expressed in GC-NR HTM cells. Two of these miRNAs associated with GC-NR (miR486-3p and miR-320a) were previously identified in the aqueous humor of glaucoma patients. In the glaucomatous aqueous humor, miR-486-3p was up-regulated but found to be down-regulated in GC-NR HTM cells [28]. Conversely, miR-320a, which was down-regulated in the aqueous humor of POAG patients, was up-regulated in GC-NR HTM cells in the present study [56]. A number of DEMIRs have been identified in HTM cells in response to various stressors such as senescence, cyclic mechanical stress, and oxidative stress [33,34,35,36,48]. Only one of these DEMIRs was identified in the current study (miR-483-3p) and was a common miRNA between GC-R and GC-NR HTM cells [57]. Mir-483-3p is known to affect the Wnt/β-catenin, the TGF-β, and the TP53 signaling pathways by targeting several genes such as CTNNB1, SMAD4, IGF1, and BBC3 [58]. In HTM cells, the expression of miR-483-3p was reduced under H_2_O_2_-induced oxidative stress, and the over-expression of 483-3p inhibited the expression of ECM proteins such as fibronectin, laminin, and collagen by targeting TGFβ2/SMAD4 signaling [57]. However, the role of miR-483-3p in steroid-induced OHT/glaucoma is not clear, which warrants further investigation.

The unique DEMIR signature between GC-R and GC-NR HTM cells provides an opportunity to identify the molecular mechanisms driving GC-responsiveness and the development of SI-OHT and glaucoma. Uniquely, miR-124-3p was significantly down-regulated in GC-R HTM cells when compared to GC-NR HTM cells. How this affects GC-responsiveness and alterations in the TM physiology is currently not known but miR124-3p can negatively regulate the expression of GR by repressing the expression of the GC receptor (NR3C1) [59,60]. The expression of miR-124-3p and hence repression of NR3C1 is mediated via SMAD4 signaling; SMAD4 is a direct target of miR-483-3p [57,61]. Elevated levels of miR-124-3p in the brain of rats and mice were associated with decreased GR levels and GC sensitivity [60], and so the potentially reduced miR-124-3p in GC-R HTM cells may suggest a role for miR-124-3p in the regulation of GR levels and GC sensitivity in the trabecular meshwork. Interestingly, miR-124-3p also modulates the GR function indirectly by targeting phosphor-diesterase 4B or 11β-hydroxysteroid dehydrogenase 1 (11β-HSD1) [62,63]. 11β-HSD1 converts cortisone and cortisol and elevated levels of cortisol have been found in patients with POAG and the inhibitors of 11β-HSD1 reduced IOP in glaucoma patients [64,65,66]; however, the exact role of 11β-HSD1 in steroid-induced glaucoma is not completely understood. Further studies are warranted to understand the role of miR124-3p in differential GC responsiveness and SI-OHT and glaucoma.

Our pathway analysis of the DEMIRs revealed several significant pathways found in both GC-R and GC-NR HTM cells (Supplementary Table S4a–e). When considering differential effects on pathway enrichment axon guidance signaling was significantly up-regulated pathway in GC-R (*p* = 0.03) and significantly down-regulated (*p* = 1.5 × 10^−5^) in GC-NR HTM cells. Interestingly, GCs are shown to have a potential role in nervous system, including stress response in neurons, synthesis of neurotransmitters, neuronal survival, and differentiation [67,68,69,70]; however, the importance of axon guidance signaling pathway in HTM cells needs further investigation.

The relaxin signaling pathway was significantly down-regulated (*p* = 0.03) in GC-R HTM cells and conversely up-regulated in GC-NR cells. Relaxin is a polypeptide hormone produced by the corpus luteum and the decidua in females and by the prostate in males [71]. Traditionally, this hormone was associated with parturition during pregnancy by relaxing collagen fibers in the pelvic region [72]. Relaxin mediates its pharmacological action by activating a group of seven transmembrane G-protein coupled receptors (GPCRs): relaxin family peptide receptors. Apart from its role in pregnancy, relaxin is produced in many tissues in mammals where it has vasodilatory and anti-fibrotic effects [73]. In the eye, the IOP lowering property of relaxin was first demonstrated by Paterson et al., 1963 following intra-mascular injection of relaxin in humans [74]. RXFP1 is expressed in sections of the anterior segment of the eye where it has been localized to the uveal, corneoscleral, and cribriform meshwork and Schlemm’s canal endothelium, suggesting its role in regulating outflow facility and IOP homeostasis [71]. Furthermore, of relevance to TM changes in response to glucocorticoids, pre-clinical studies suggest the potential use of relaxin as an anti-fibrotic molecule in several organs, such as the lung, skin, kidney, heart, and liver [75]. Given that relaxin signaling was down-regulated in GC-R HTM cells and relaxin mediates an anti-fibrotic activity through its cognate receptor (RXFP1) [76], the role of relaxin in SI-OHT and glaucoma requires further studies.

Our integrative miRNA-mRNA analysis of both in silico and our own RNA sequencing data from paired TM donor cells with known GC responsiveness [20] identified several potential miRNA-mRNA interactions highlighting GC-responsive and non-responsive molecular regulators. For example, in GC-R HTM cells, the up-regulation of miR 2114-3p was associated with the down-regulation of several target genes such as NDP, GJB2, RAMP3, PDZK1P1, RTN4RL1, DRAXIN, KRT6, S100A14, CHRNA4, and RIC3. Whereas the down-regulation of miR-124-3p in the GC-R HTM cells was associated with the up-regulation of the transcription factor KLF-15. In the GC-NR group, miR-486-3p, miR-181b-1-3p, and miR-10396b-5p were found to be molecular regulators in mediating GC-non-responsiveness by targeting several mRNA genes.

In conclusion, this is the first study that identified a unique miRNA signature between GC-R and GC-NR HTM cells using small RNA sequencing. Utilizing the human organ-cultured anterior segment ex vivo model enabled us to identify the induction of GC-OHT after DEX treatment and so classify HTM cells based on GC responsiveness: GC-R and GC-NR. Ingenuity Pathway Analysis identified enriched pathways and biological processes associated with differential glucocorticoid (GC) responsiveness in HTM cells. Integrative analysis of miRNA-mRNA of the same set of HTM cells revealed several molecular regulators for GC non-responsiveness. Previous mRNA data of the same set of HTM cells allowed us to perform the integrative analysis of miRNA-mRNA and revealed several molecular regulators for GC non-responsiveness. Additional functional studies of these molecular regulators are warranted to validate their role in differential GC responsiveness. Understanding the role of miRNAs in GC responsiveness raises the possibility of new molecular targets for the management of steroid-OHT/glaucoma.

## Figures and Tables

**Table 1:** List of overlapping and up/down-regulated miRNAs from Group #3, #4 (GC-R), and #5 (GC-NR).

**List of Overlapping miRNAs from Group #3**							
**miRNA**	**Group #1**			**Group #2**			
	**logFC**	**logCPM**	***p*-Value**	**logFC**	**logCPM**		***p*-Value**
hsa-miR-675-3p	4.86	0.11	8.01 × 10^−4^	3.63	2.61		0.01
hsa-miR-483-3p	4.61	2.88	1.01 × 10^−4^	4.05	3.36		5.60 × 10^−5^
hsa-miR-675-5p	4.59	1.20	2.88 × 10^−4^	3.67	3.14		0.00
hsa-miR-483-5p	4.31	0.82	1.16 × 10^−4^	4.65	1.20		4.42 × 10^−6^
hsa-miR-5690	3.61	−1.26	0.00	2.15	−0.79		0.02
hsa-miR-6842-3p	1.50	1.33	0.02	1.50	2.08		0.01
hsa-miR-335-3p	−3.03	8.41	1.01 × 10^−4^	−1.93	8.56		0.00
**List of Up/Down-regulated miRNAs from Group #4**							
**Up-regulated miRNAs**				**Down-regulated miRNAs**			
**miRNA**	**logFC**	**logCPM**	***p*-Value**	**miRNA**	**logFC**	**logCPM**	***p*-Value**
hsa-miR-2114-3p	6.07	−1.16	4.25 × 10^−4^	hsa-miR-335-5p	−1.71	6.75	0.02
hsa-miR-2114-5p	3.17	−0.83	0.02	hsa-miR-549a-5p	−2.15	0.89	0.02
				hsa-miR-7151-3p	−3.08	−0.31	0.03
				hsa-miR-124-3p	−6.21	−0.81	0.01
**List of Up/Down-regulated miRNAs from Group #5**							
**Up-regulated miRNAs**				**Down-regulated miRNAs**			
**miRNA**	**logFC**	**logCPM**	***p*-Value**	**miRNA**	**logFC**	**logCPM**	***p*-Value**
hsa-miR-4485-5p	4.73	1.01	2.89 × 10^−4^	hsa-miR-181b-2-3p	−1.21	0.85	0.04
hsa-miR-12136	4.39	7.32	0.00	hsa-miR-486-3p	−1.34	2.38	0.05
hsa-miR-4328	4.11	−0.38	0.02	hsa-miR-6853-3p	−1.80	−0.69	0.03
hsa-miR-320a-5p	3.47	1.78	0.00	hsa-miR-550a-3p	−2.06	−0.14	0.03
hsa-miR-4485-3p	3.07	3.57	0.00	hsa-miR-9-3p	−4.88	−1.33	0.02
hsa-miR-10396b-5p	2.66	−0.69	0.01				
hsa-miR-10396a-5p	2.60	−0.72	0.01				
hsa-miR-3195	2.44	−0.73	0.04				
hsa-miR-1246	2.24	1.01	0.01				

Note: Group #3: DE miRNAs that overlapping between Group #1 and Group #2; Group #4: uniquely expressed DE miRNAs of GC-R HTM cells (Group #1 minus Group #3); Group#5: uniquely expressed DEGs of GC-NR HTM cells (Group #2 minus Group #3).

**Table 2:** Showing the target mRNAs that negatively correlated with DEMIRs in Group#3–#5.

**(i)** **Target mRNAs That Negatively Correlated with DEMIRs in Group#3**		
**ID of DEMIRs**	**Count of Target mRNAs**	**ID of Target mRNAs**
hsa-miR-483-5p	1	RGS16
hsa-miR-6842-3p	1	NGFR
**(ii)** **Target mRNAs that negatively correlated with DEMIRs in Group#4**		
hsa-miR-124-3p	1	KLF15
hsa-miR-2114-3p	11	CHRNA4, DRAXIN, GJB2, JCHAIN, KRT8, NDP, PDZK1IP1, RAMP3, RIC3, RTN4RL1, S100A14
hsa-miR-2114-5p	2	CXADR, NTF4
hsa-miR-335-5p	1	MAP1LC3C
**(iii)** **Target mRNAs that negatively correlated with DEMIRs in Group#5**		
hsa-miR-10396b-5p	4	CHAC1, INA, NMNAT2, UNC5B
hsa-miR-181b-2-3p	1	ALKAL2
hsa-miR-320a-5p	1	CHAC1
hsa-miR-4328	1	SLC24A2
hsa-miR-486-3p	4	DUSP5, LGI3, MREG, SOX13
hsa-miR-10396a-5p	3	CHAC1, IL32, SLC7A5

## Data Availability

Data Access: The raw miRNA sequencing data of HTM cells from each human donor eye used in the present study have been deposited publicly in NCBI-SRA under the BioProject PRJNA982055 (https://www.ncbi.nlm.nih.gov/sra/PRJNA982055, accessed on 9 June 2023). Code Availability: The bioinformatics In-house pipeline used for mRNA sequencing data analysis in the present study have been submitted to GitHub in shell script (https://github.com/SenthilKumariLab/mRNA-seq-Analysis-Pipeline.git, accessed on 14 May 2021).

## Supplementary Materials

The following supporting information can be downloaded at: https://www.mdpi.com/article/10.3390/genes14112012/s1, Table S1: Characteristics of Human Trabecular Meshwork Cells Used for the Present Study; Table S2:Alignment Statistics of miRNA Sequencing Data; Table S3a. List of Up/Down-regulated miRNAs from Group #1; Table S3b. List of Up/Down-regulated miRNAs from Group #2; Table S4a. List of Enriched Pathways from DE miRNAs of Group #1; Table S4b. List of Enriched Pathways from DE miRNAs of Group #2; Table S4c. List of Enriched Pathways from DE miRNAs of Group #3; Table S4d. List of Enriched Pathways from DE miRNAs of Group #4; Table S4e. List of Enriched Pathways from DE miRNAs of Group #5; Table S5. List of miRNAs Selected from miRNA sequencing for Validation by qPCR; Table S6 showing the overlapping biological processes in ‘Target MRNA List 1 & 2’ from Group#3–#5; Supplementary File S1 List S1A: mRNA target prediction; Supplementary File S1 List S1B: Pathway analysis for target mRNA List 1A; Supplementary File S1 List S1C: Biological process analysis for mRNA List 1A; Supplementary File S2 List S2A: mRNA target prediction; Supplementary File S2 List S2B: Pathway analysis for target mRNA List2A; Supplementary File S2 List S2C: Pathway analysis of mRNA target using IPA software; Supplementary File S2 List S2D: Comparative anal pathway of target mRNA_group #3–#5; Supplementary File S2 List S2E: Biological process analysis for mRNA List2A.

## References

[1] Gordon D.M. (1955). Hormonal-steroid therapy in ocular inflammations. Seminar.

[2] Vajaranant T.S., Price M.O., Price F.W., Gao W., Wilensky J.T., Edward D.P. (2009). Visual acuity and intraocular pressure after Descemet’s stripping endothelial keratoplasty in eyes with and without preexisting glaucoma. Ophthalmology.

[3] Jonas J.B., Akkoyun I., Kreissig I., Degenring R.F. (2005). Diffuse diabetic macular oedema treated by intravitreal triamcinolone acetonide: A comparative, non-randomised study. Br. J. Ophthalmol..

[4] Gregori N.Z., Rosenfeld P.J., Puliafito C.A., Flynn H.W., Lee J.E., Mavrofrides E.C., Smiddy W.E., Murray T.G., Berrocal A.M., Scott I.U. (2006). One-year safety and efficacy of intravitreal triamcinolone acetonide for the management of macular edema secondary to central retinal vein occlusion. Retina.

[5] Schwartz S.G., Flynn H.W., Beer P. (2010). Intravitreal Triamcinolone Acetonide Use in Diabetic Macular Edema: Illustrative Cases. Ophthalmic Surg. Lasers Imaging.

[6] Lowder C., Belfort R., Lightman S., Foster C.S., Robinson M.R., Schiffman R.M., Li X.Y., Cui H., Whitcup S.M. (2011). Dexamethasone intravitreal implant for noninfectious intermediate or posterior uveitis. Arch. Ophthalmol..

[7] Aref A.A., Scott I.U., Oden N.L., Ip M.S., Blodi B.A., Van Veldhuisen P.C. (2015). Incidence, Risk Factors, and Timing of Elevated Intraocular Pressure After Intravitreal Triamcinolone Acetonide Injection for Macular Edema Secondary to Retinal Vein Occlusion: SCORE Study Report 15. JAMA Ophthalmol..

[8] Kiddee W., Trope G.E., Sheng L., Beltran-Agullo L., Smith M., Strungaru M.H., Baath J., Buys Y.M. (2013). Intraocular pressure monitoring post intravitreal steroids: A systematic review. Surv. Ophthalmol..

[9] Campochiaro P.A., Brown D.M., Pearson A., Chen S., Boyer D., Ruiz-Moreno J., Garretson B., Gupta A., Hariprasad S.M., Bailey C. (2012). Sustained delivery fluocinolone acetonide vitreous inserts provide benefit for at least 3 years in patients with diabetic macular edema. Ophthalmology.

[10] Chin E.K., Almeida D.R.P., Velez G., Xu K., Peraire M., Corbella M., Elshatory Y.M., Kwon Y.H., Gehrs K.M., Boldt H.C. (2017). Ocular Hypertension after Intravitreal Dexamethasone (OZURDEX) Sustained-Release implant. Retina.

[11] Becker B., Mills D.W. (1963). Corticosteroids and Intraocular Pressure. Arch. Ophthalmol..

[12] Bartlett J.D., Woolley T.W., Adams C.M. (1993). Identification of High Intraocular Pressure Responders to Topical Ophthalmic Corticosteroids. J. Ocul. Pharmacol. Ther..

[13] Lewis J.M., Priddy T., Judd J., Gordon M.O., Kass M.A., Kolker A.E., Becker B. (1988). Intraocular Pressure Response to Topical Dexamethasone as a Predictor for the Development of Primary Open-Angle Glaucoma. Am. J. Ophthalmol..

[14] Armaly M.F. (1963). Effect of Corticosteroids on Intraocular Pressure and Fluid Dynamics: I. The Effect of Dexamethasone in the Normal Eye. Arch. Ophthalmol..

[15] Becker B. (1965). Intraocular Pressure Response to Topical Corticosteroids. Investig. Ophthalmol. Vis. Sci..

[16] Feroze K.B., Zeppieri M., Khazaeni L. (2023). Steroid-Induced Glaucoma. StatPearls.

[17] Gerzenstein S.M., Pletcher M.T., Cervino A.C.L., Tsinoremas N.F., Young B., Puliafito C.A., Fini M.E., Schwartz S.G. (2008). Glucocorticoid Receptor Polymorphisms and Intraocular Pressure Response to Intravitreal Triamcinolone Acetonide. Ophthalmic Genet..

[18] Fingert J.H., Alward W.L., Wang K., Yorio T., Clark A.F. (2010). Assessment of SNPs associated with the human glucocorticoid receptor in primary open-angle glaucoma and steroid responders. Mol. Vis..

[19] Haribalaganesh R., Gowri Priya C., Sharmila R., Krishnadas S., Muthukkaruppan V., Willoughby C.E., Senthilkumari S. (2021). Assessment of differential intraocular pressure response to dexamethasone treatment in perfusion cultured Indian cadaveric eyes. Sci. Rep..

[20] Kathirvel K., Haribalaganesh R., Krishnadas R., Muthukkaruppan V., Willoughby C.E., Bharanidharan D., Senthilkumari S. (2022). A Comparative Genome-Wide Transcriptome Analysis of Glucocorticoid Responder and Non-Responder Primary Human Trabecular Meshwork Cells. Genes.

[21] Guo H., Ingolia N.T., Weissman J.S., Bartel D.P. (2010). Mammalian microRNAs predominantly act to decrease target mRNA levels. Nature.

[22] Mitchell P.S., Parkin R.K., Kroh E.M., Fritz B.R., Wyman S.K., Pogosova-Agadjanyan E.L., Peterson A., Noteboom J., O’Briant K.C., Allen A. (2008). Circulating microRNAs as stable blood-based markers for cancer detection. Proc. Natl. Acad. Sci. USA.

[23] Kumar S., Reddy P.H. (2018). MicroRNA-455-3p as a potential biomarker for Alzheimer’s disease: An update. Front. Aging Neurosci..

[24] Liu C., Marioni R.E., Hedman A.K., Pfeiffer L., Tsai P.C., Reynolds L.M., Just A.C., Duan Q., Boer C.G., Tanaka T. (2018). A DNA methylation biomarker of alcohol consumption. Mol. Psychiatry.

[25] Kim M., Zhang X. (2019). The Profiling and Role of miRNAs in Diabetes Mellitus. J. Diabetes Clin. Res..

[26] Hackler L., Wan J., Swaroop A., Qian J., Zack D.J. (2010). MicroRNA profile of the developing mouse retina. Investig. Ophthalmol. Vis. Sci..

[27] Busskamp V., Krol J., Nelidova D., Daum J., Szikra T., Tsuda B., Jüttner J., Farrow K., Scherf B.G., Alvarez C.P.P. (2014). MiRNAs 182 and 183 Are Necessary to Maintain Adult Cone Photoreceptor Outer Segments and Visual Function. Neuron.

[28] Tanaka Y., Tsuda S., Kunikata H., Sato J., Kokubun T., Yasuda M., Nishiguchi K.M., Inada T., Nakazawa T. (2014). Profiles of extracellular miRNAs in the aqueous humor of glaucoma patients assessed with a microarray system. Sci. Rep..

[29] Jayaram H., Phillips J.I., Lozano D.C., Choe T.E., Cepurna W.O., Johnson E.C., Morrison J.C., Gattey D.M., Saugstad J.A., Keller K.E. (2017). Comparison of microRNA expression in aqueous humor of normal and primary open-angle glaucoma patients using PCR arrays: A pilot study. Investig. Ophthalmol. Vis. Sci..

[30] Hubens W.H.G., Krauskopf J., Beckers H.J.M., Kleinjans J.C.S., Webers C.A.B., Gorgels T.G.M.F. (2021). Small RNA sequencing of aqueous humor and plasma in patients with primary open-angle glaucoma. Investig. Ophthalmol. Vis. Sci..

[31] Kosior-Jarecka E., Czop M., Gasińska K., Wróbel-Dudzińska D., Zalewski D.P., Bogucka-Kocka A., Kocki J., Żarnowski T. (2021). MicroRNAs in the aqueous humor of patients with different types of glaucoma. Graefe’s Arch. Clin. Exp. Ophthalmol..

[32] Polansky J.R., Kurtz R.M., Fauss D.J., Kim R.Y., Bloom E. (1991). In Vitro Correlates of Glucocorticoid Effects on Intraocular Pressure. Glaucoma Update IV.

[33] Li G., Luna C., Qiu J., Epstein D.L., Gonzalez P. (2010). Modulation of inflammatory markers by miR-146a during replicative senescence in trabecular meshwork cells. Investig. Ophthalmol. Vis. Sci..

[34] Zhao J., Du X., Wang M., Yang P., Zhang J. (2019). Salidroside mitigates hydrogen peroxide-induced injury by enhancement of microRNA-27a in human trabecular meshwork cells. Artif. Cells, Nanomed. Biotechnol..

[35] Shen W., Wang C., Huang B. (2020). Oxidative Stress-Induced circHBEGF Promotes Extracellular Matrix Production via Regulating miR-646/EGFR in Human Trabecular Meshwork Cells. Oxid. Med. Cell. Longev..

[36] Youngblood H., Cai J., Drewry M.D., Helwa I., Hu E., Liu S., Yu H., Mu H., Hu Y., Perkumas K. (2020). Expression of mRNAs, miRNAs, and lncRNAs in human trabecular meshwork cells upon mechanical stretch. Investig. Ophthalmol. Vis. Sci..

[37] Clayton S.A., Jones S.W., Kurowska-Stolarska M., Clark A.R. (2018). The role of microRNAs in glucocorticoid action. J. Biol. Chem..

[38] Yemanyi F., Vranka J., Raghunathan V.K. (2020). Glucocorticoid-induced cell-derived matrix modulates transforming growth factor β2 signaling in human trabecular meshwork cells. Sci. Rep..

[39] Whirledge S., DeFranco D.B. (2018). Glucocorticoid signaling in health and disease: Insights from tissue-Specific GR knockout mice. Endocrinology.

[40] Kathirvel K., Karen L., Haribalaganesh R., Krishnadas R., Muthukkaruppan V., Lane B., Simpson D.A., Goljanek-Whysall K., Sheridan C., Bharanidharan D. (2022). Short and long-term effect of dexamethasone on the transcriptome profile of primary human trabecular meshwork cells in vitro. Sci. Rep..

[41] Stamer W.D., Seftor R.E.B., Williams S.K., Samaha H.A.M., Snyder R.W. (1995). Isolation and culture of human trabecular meshwork cells by extracellular matrix digestion. Curr. Eye Res..

[42] Ashwinbalaji S., Senthilkumari S., Gowripriya C., Krishnadas S., Gabelt B.A.T., Kaufman P.L., Muthukkaruppan V. (2018). SB772077B, A New Rho Kinase Inhibitor Enhances Aqueous Humour Outflow Facility in Human Eyes. Sci. Rep..

[43] Keller K.E., Bhattacharya S.K., Borrás T., Brunner T.M., Chansangpetch S., Clark A.F., Dismuke W.M., Du Y., Elliott M.H., Ethier C.R. (2018). Consensus recommendations for trabecular meshwork cell isolation, characterization and culture. Exp. Eye Res..

[44] Fang Z., Liu X., Peltz G. (2023). GSEApy: A comprehensive package for performing gene set enrichment analysis in Python. Bioinformatics.

[45] Fini M.E., Schwartz S.G., Gao X., Jeong S., Patel N., Itakura T., Price M.O., Price F.W., Varma R., Stamer W.D. (2017). Steroid-induced ocular hypertension/glaucoma: Focus on pharmacogenomics and implications for precision medicine. Prog. Retin. Eye Res..

[46] Clark A.F., Wordinger R.J. (2009). The role of steroids in outflow resistance. Exp. Eye Res..

[47] Liesenborghs I., Eijssen L.M.T., Kutmon M., Gorgels T.G.M.F., Evelo C.T., Beckers H.J.M., Webers C.A.B., Schouten J.S.A.G. (2020). The Molecular Processes in the Trabecular Meshwork After Exposure to Corticosteroids and in Corticosteroid-Induced Ocular Hypertension. Investig. Opthalmol. Vis. Sci..

[48] Li G., Luna C., Qiu J., Epstein D.L., Gonzalez P. (2009). Alterations in microRNA expression in stress-induced cellular senescence. Mech. Ageing Dev..

[49] Clark A.F., Wilson K., McCartney M.D., Miggans S.T., Kunkle M., Howe W. (1994). Glucocorticoid-induced formation of cross-linked actin networks in cultured human trabecular meshwork cells. Investig. Ophthalmol. Vis. Sci..

[50] Clark A.F., Wilson K., De Kater A.W., Allingham R.R., McCartney M.D. (1995). Dexamethasone-induced ocular hypertension in perfusion-cultured human eyes. Investig. Ophthalmol. Vis. Sci..

[51] Wordinger R.J., Clark A.F., Agarwal R., Lambert W., Wilson S.E. (1999). Expression of alternatively spliced growth factor receptor isoforms in the human trabecular meshwork. Investig. Ophthalmol. Vis. Sci..

[52] Zhou L., Li Y., Yue B.Y. (1998). Glucocorticoid effects on extracellular matrix proteins and integrins in bovine trabecular meshwork cells in relation to glaucoma. Int. J. Mol. Med..

[53] Tane N., Dhar S., Roy S., Pinheiro A., Ohira A., Roy S. (2007). Effect of excess synthesis of extracellular matrix components by trabecular meshwork cells: Possible consequence on aqueous outflow. Exp. Eye Res..

[54] Zhang X., Ognibene C.M., Clark A.F., Yorio T. (2007). Dexamethasone inhibition of trabecular meshwork cell phagocytosis and its modulation by glucocorticoid receptor β. Exp. Eye Res..

[55] Stamer W.D., Clark A.F. (2017). The many faces of the trabecular meshwork cell. Exp. Eye Res..

[56] Drewry M.D., Challa P., Kuchtey J.G., Navarro I., Helwa I., Hu Y., Mu H., Stamer W.D., Kuchtey R.W., Liu Y. (2018). Differentially expressed microRNAs in the aqueous humor of patients with exfoliation glaucoma or primary open-angle glaucoma. Hum. Mol. Genet..

[57] Shen W., Han Y., Huang B., Qi Y., Xu L., Guo R., Wang X., Wang J. (2015). MicroRNA-483-3p inhibits extracellular matrix production by targeting smad4 in human trabecular meshwork cells. Investig. Ophthalmol. Vis. Sci..

[58] Pepe F., Visone R., Veronese A. (2018). The Glucose-Regulated MiR-483-3p Influences Key Signaling Pathways in Cancer. Cancers.

[59] Vreugdenhil E., Verissimo C.S.L., Mariman R., Kamphorst J.T., Barbosa J.S., Zweers T., Champagne D.L., Schouten T., Meijer O.C., De Ron Kloet E. (2009). MicroRNA 18 and 124a down-regulate the glucocorticoid receptor: Implications for glucocorticoid responsiveness in the brain. Endocrinology.

[60] Wang S.S., Mu R.H., Li C.F., Dong S.Q., Geng D., Liu Q., Yi L.T. (2017). microRNA-124 targets glucocorticoid receptor and is involved in depression-like behaviors. Prog. Neuro-Psychopharmacol. Biol. Psychiatry.

[61] Liu K., Zhang X., Wei W., Liu X., Tian Y., Han H., Zhang L., Wu W., Chen J. (2019). Myostatin/smad4 signaling-mediated regulation of mir-124-3p represses glucocorticoid receptor expression and inhibits adipocyte differentiation. Am. J. Physiol. Endocrinol. Metab..

[62] Kim J., Jeong D., Nam J., Aung T.N., Gim J.A., Park K.U., Kim S.W. (2015). MicroRNA-124 regulates glucocorticoid sensitivity by targeting phosphodiesterase 4B in diffuse large B cell lymphoma. Gene.

[63] Xu J., Wang R., Liu Y., Liu D., Jiang H., Pan F. (2017). FKBP5 and specific microRNAs via glucocorticoid receptor in the basolateral amygdala involved in the susceptibility to depressive disorder in early adolescent stressed rats. J. Psychiatr. Res..

[64] Rauz S., Cheung C.M.G., Wood P.J., Coca-Prados M., Walker E.A., Murray P.I., Stewart P.M. (2003). Inhibition of 11β-hydroxysteroid dehydrogenase type of 1 lowers intraocular pressure in patients with ocular hypertension. QJM-Mon. J. Assoc. Physicians.

[65] Choi K.J., Na Y.J., Park S.B., Jung W.H., Sung H.R., Kim K.Y. (2017). Carbenoxolone prevents chemical eye ischemia-reperfusion-induced cell death via 11β-hydroxysteroid dehydrogenase type 1 inhibition. Pharmacol. Res..

[66] Choi K.J., Na Y.J., Jung W.H., Park S.B., Kang S., Nam H.J., Ahn J.H., Kim K.Y. (2019). Protective effect of a novel selective 11β-HSD1 inhibitor on eye ischemia-reperfusion induced glaucoma. Biochem. Pharmacol..

[67] McEwen B.S., De Kloet E.R., Rostene W. (1986). Adrenal steroid receptors and actions in the nervous system. Physiol. Rev..

[68] Gould E., Tanapat P. (1999). Stress and hippocampal neurogenesis. In Proceedings of the Biological Psychiatry. Biol. Psychiatry.

[69] Glick R.D., Medary I., Aronson D.C., Scotto K.W., Swendeman S.L., La Quaglia M.P. (2000). The effects of serum depletion and dexamethasone on growth and differentiation of human neuroblastoma cell lines. J. Pediatr. Surg..

[70] Wang W.Y., Pan L., Su S.C., Quinn E.J., Sasaki M., Jimenez J.C., MacKenzie I.R.A., Huang E.J., Tsai L.H. (2013). Interaction of FUS and HDAC1 regulates DNA damage response and repair in neurons. Nat. Neurosci..

[71] Zloto O., Skaat A., Fabian I.D., Rosner M., Ziv H., Leshno A., Melamed S. (2020). The distribution of relaxin receptors in the anterior segment of primary open-angle glaucoma patients. Indian J. Ophthalmol..

[72] Hisaw F.L. (1926). Experimental relaxation of the pubic ligament of the guinea pig. Proc. Soc. Exp. Biol. Med..

[73] Bathgate R., Dschietzig T., Gundlach A.L., Halls M., Summers R. (2021). Relaxin family peptide receptors in GtoPdb v.2021.3. IUPHAR/BPS Guide Pharmacol. CITE.

[74] Paterson G.D., Miller S.J. (1963). Hormonal influence in simple Glaucoma. A Preliminary report. Br. J. Ophthalmol..

[75] Chen T.Y., Li X., Hung C.H., Bahudhanapati H., Tan J., Kass D.J., Zhang Y. (2020). The relaxin family peptide receptor 1 (RXFP1): An emerging player in human health and disease. Mol. Genet. Genom. Med..

[76] Ng H.H., Shen M., Samuel C.S., Schlossmann J., Bennett R.G. (2019). Relaxin and extracellular matrix remodeling: Mechanisms and signaling pathways. Mol. Cell. Endocrinol..

